# The Immune Underpinnings of Barrett’s-Associated Adenocarcinogenesis: a Retrial of Nefarious Immunologic Co-Conspirators

**DOI:** 10.1016/j.jcmgh.2022.01.023

**Published:** 2022-02-03

**Authors:** Louisa Tambunting, Dermot Kelleher, Shane Patrick Duggan

**Affiliations:** 1 Life Science Institute, University of British Columbia, Vancouver, Canada; 2 Division of Gastroenterology, Department of Medicine, Faculty of Medicine, University of British Columbia, Vancouver, Canada

**Keywords:** Esophagus, Metaplasia, Dysplasia, Barrett’s esophagus, Esophageal adenocarcinoma, Gastro-esophageal reflux disease, Cytokine, Immunology, Inflammation, Interleukin 1β, Interleukin 6, Leukemia Inhibitory Factor, Signal Transduction and Activator of Transcription 3, Tumor Necrosis Factor α, BE, Barrett’s esophagus, COX2, cyclooxygenase-2, C1q, complement component 1q, EAC, esophageal adenocarcinoma, EoE, eosinophilic esophagitis, ESCC, esophageal squamous cell carcinoma, GATA6, GATA binding protein 6, GDF, growth differentiation factor, GEJ, gastroesophageal junction, GERD, gastroesophageal reflux disease, HGD, high-grade dysplasia, IFN-γ, interferon γ, IκB, nuclear factor of kappa light polypeptide gene enhancer in B-cells inhibitor, IL, interleukin, JAK3, Janus kinase 3, LIF, leukemia inhibitory factor, MDSC, myeloid-derived suppressor cell, NF-κB, nuclear factor-κB, NK, natural killer, PGE2, prostaglandin E2, PPI, proton pump inhibitor, SCJ, squamocolumnar junction, SMAD, small mothers against decapentaplegic, STAT, signal transduction and activator of transcription, SYK, Spleen tyrosine kinase, TAM, tumor-associated macrophage, TGFβ, transforming growth factor β, Th, T-helper cell, TME, tumor microenvironment, TNF, tumor necrosis factor, TNFR, tumor necrosis factor receptor

## Abstract

There is no doubt that chronic gastroesophageal reflux disease increases the risk of esophageal adenocarcinoma (EAC) by several fold (odds ratio, 6.4; 95% CI, 4.6–9.1), and some relationships between reflux disease–mediated inflammation and oncogenic processes have been explored; however, the precise interconnections between the immune response and genomic instabilities underlying these pathologic processes only now are emerging. Furthermore, the precise cell of origin of the precancerous stages associated with EAC development, Barrett’s esophagus, be it cardia resident or embryonic remnant, may shape our interpretation of the likely immune drivers. This review integrates the current collective knowledge of the immunology underlying EAC development and outlines a framework connecting proinflammatory pathways, such as those mediated by interleukin 1β, tumor necrosis factor α, leukemia inhibitory factor, interleukin 6, signal transduction and activator of transcription 3, nuclear factor-κB, cyclooxygenase-2, and transforming growth factor β, with oncogenic pathways in the gastroesophageal reflux disease–Barrett’s esophagus–EAC cancer sequence. Further defining these immune and molecular railroads may show a map of the routes taken by gastroesophageal cells on their journey toward EAC tumor phylogeny. The selective pressures applied by this immune-induced journey likely impact the phenotype and genotype of the resulting oncogenic destination and further exploration of lesser-defined immune drivers may be useful in future individualized therapies or enhanced selective application of recent immune-driven therapeutics.

Esophageal adenocarcinoma (EAC) is clinically relevant as a result of its increasing disease demographic, high mortality rate (<20% 5-year survival), and lack of effective and early prescreening methods. Although EAC has affected mainly Western nations, the global spread of a high-calorie, cholesterol-rich diet has increased the occurrence of both abdominal obesity and gastroesophageal reflux disease (GERD): 2 factors that increase the risk of EAC by several-fold.^1^ For example, demographics with traditionally low EAC to esophageal squamous cell carcinoma (ESCC) incidence, such as those in several East Asian countries, have seen a progressive increase in the EAC/ESCC ratio that is parallel to the increase in obesity.^1^ Thus, the study of EAC pathogenesis and its treatment remains a pressing issue. Genome-wide association studies have defined polymorphic variation associated with GERD, Barrett’s esophagus (BE), and EAC development.^2^, ^3^, ^4^ These data recently were used to show that one of the most significantly genetically correlated phenotypes with GERD was BE/EAC, along with depression and education, using Linkage disequilibrium (LD) score regression analyses.^2^^,^^5^

The transient yet long-term exposure of esophageal tissue by gastric contents resulting from GERD, in addition to the accompanying immune response, has been theorized as a key developmental driver of BE, a potentially premalignant condition highly associated with EAC. As a type of intestinal metaplasia, BE is characterized by the replacement of stratified squamous epithelium with simple columnar epithelium that overlies mucous-secreting glands. Evidence from animal models, human cell lines, and patient-derived tissues has shown several potential cellular origins of BE. These include esophageal progenitor cells from either the basal squamous epithelium or in ductal cells of submucosal glands, squamocolumnar junction, gastric cardia, or bone marrow^6^, ^7^, ^8^, ^9^, ^10^, ^11^, ^12^, ^13^, ^14^, ^15^, ^16^, ^17^, ^18^, ^19^, ^20^, ^21^, ^22^; however, despite several decades of vigorous scientific inquiry, the origin of BE cells remains unknown. Regardless of its source, BE has been shown to differentiate into dysplastic populations over time as the highly inflammatory, GERD-induced environment ultimately selects for the expansion of highly proliferative and apoptotic-resistant clones. As the accumulation of precancerous mutations, such as loss of Tumor Protein P53 (TP53) and Cyclin dependent kinase inhibitor 2A (CDKN2A), persist in certain dysplastic populations, these populations may undergo tumorigenesis and subsequent metastasis.^23^, ^24^, ^25^ Metaplastic transformation of the gastroesophageal junction is linked consistently with the reflux of acidified bile acid and much of the initial research focused on the ability of acidified bile acid to induce the expression of intestine phenotype-associated genes such as mucins or keratins. Although the caustic damage from sustained acid/bile acid reflux plays a significant part in the selection of precancerous BE cells, the delayed immune response that follows injury may play a much more significant role in shaping the molecular landscape underlying GERD-BE-EAC transitions. For example, Souza et al^26^ showed that esophageal cell lines exposed to acidic bile did not show immediate caustic injury, but rather a delayed inflammatory immune response. In addition, many of these inflammatory factors, mostly cytokines, may act as the molecular crossroads that connect the immunologic, metaplastic, and metastatic railways that form the tumorigenic path toward EAC. Of these pathways, the major molecules of interest in this review are interleukin (IL)1β, tumor necrosis factor α (TNFα), nuclear factor-κB (NF-κB), leukemia inhibitory factor (LIF), IL6, signal transduction and activator of transcription (STAT)3, cyclooxygenase-2 (COX2) and associated prostaglandins, transforming growth factor β (TGFβ), and complement proteins. This review will contextualize and integrate these pathways in chronological order from a primarily immunologic standpoint, concluding with a discussion on how these perspectives aid the development of personalized oncogenomic treatments targeting key dysregulated pathways central to the pathogenesis of EAC.

## In the Beginning: Innate IL1β Activation

**Figure 1:**
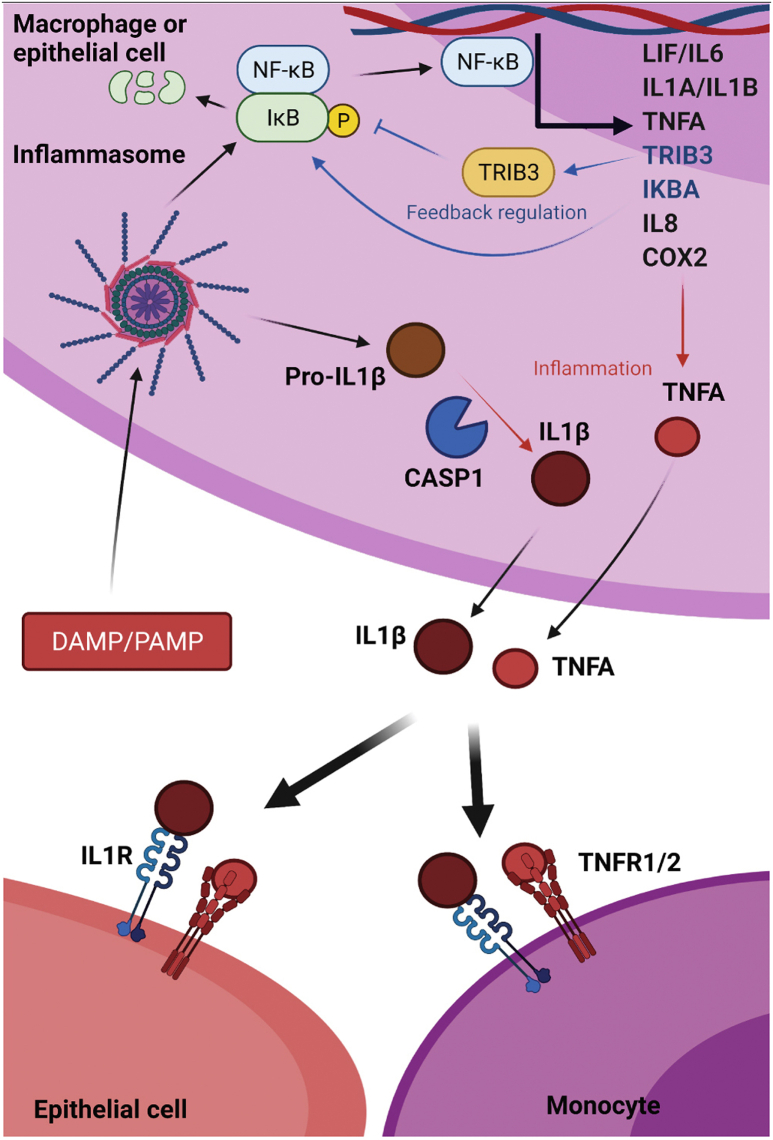
**Inflammatory starting line.** Damage-associated molecular pattern molecules (DAMP), resulting from GERD tissue damage, induce the formation and activity of the nucleotide-binding oligomerization domain–like receptor pyrin domain containing 3 (NLRP3) inflammasome and cytokine release. Pro-IL1β is cleaved by the inflammasome and caspase 1 into its active form, followed by release of both IL1β and TNFα from tissue resident monocytes and macrophages. IL1β recruits monocytes from the bone marrow— whose arrival and exposure to further IL1β, TNFα largely through TNFR2-receptor engagement, and GERD constituents—further amplifies the inflammatory response. The presence of TNFR1 and/or TNFR2 receptors dictate the specificity of the response to TNFα exposure and the induction of inflammatory cytokines, such as IL6 and IL8, to further increase TNFα messenger RNA transcription via NF-κB transcription factor activation. Exposure of esophageal epithelial cells to IL1β and TNFα results in pathway activations in a cell type and pathology-specific manner. Both exogenous IL1β and TNFα may initiate phosphorylation-dependent signaling in epithelial cells through the IκB kinase complex, resulting in phosphorylation and ubiquitination of IκBα, subsequent release of the NF-κB transcription factor, and translocation into the nucleus to induce inflammatory cytokine and prostaglandin production (IL8, IL6, LIF, and COX2). IL1β and TNFα signaling may be suppressed by the initiation of feedback regulators such as the IL1RA-receptor antagonist, IκBα replenishment, and induction of the noncanonical pseudokinase tribbles homology 3 (TRIB3), which acts to block phosphorylation of IκBα. Mounting evidence suggests tissue- and cell type–specific responses to bile acid and acid reflux, especially when comparing between normal esophageal and metaplastic tissues.^26^^,^^37^, ^38^, ^39^^,^^46^, ^47^, ^48^, ^49^, ^50^, ^51^, ^52^, ^53^, ^54^ CASP1, Caspase 1; PAMP, Pathogen-associated molecular pattern; TNFA, Tumor necrosis factor alpha.

IL1β is a proinflammatory cytokine that plays an important role in the initiation of the innate immune response.^27^, ^28^, ^29^, ^30^ Pattern recognition receptors such as Toll-like receptors, retinoic acid inducible gene-I (RIG-1) like receptors, and nucleotide-binding oligomerization domain–like receptors are important in the innate activation of IL1β.^27^, ^28^, ^29^, ^30^, ^31^ The cleavage of pro-IL1β by caspase-1 is required for the formation of active IL1β, which then may induce consequent proinflammatory IL6 and IL8 expression through NF-κB–mediated signaling (Figure 1). Dysregulated IL1β signaling, caused by genetically derived aberrations of the inflammasome complex or cellular exposure to chronic inflammatory agents, is a hallmark of several diseases such as inflammatory bowel disease, type 2 diabetes, and obesity.^27^^,^^32^, ^33^, ^34^ IL1β also has been implicated in the progression of several cancers, including gastric, pancreatic ductal adenocarcinoma, breast cancer, and lung adenocarcinoma.^27^^,^^32^, ^33^, ^34^, ^35^, ^36^ Analysis of polymorphic variation surrounding the *IL1B* gene along the esophageal disease spectrum has trended toward nonsignificant associations. Nonetheless, a histologic inflammatory gradient has been observed in BE, defined by higher levels of IL1β and IL8, and was lowest at the esophageal z-line and progressively higher toward the new squamocolumnar junction of the BE lesion distal from the gastroesophageal junction (GEJ).^37^ Further studies by Abdel-Latif et al^38^ observed that IL1β expression increased progressively, coincidental with NF-κB transcriptional activation, from esophagitis through BE-EAC. Interestingly, reflux-mediated induction of IL1β using in vitro models was higher in EAC cells than cells of squamous origins, most likely owing to differences in NF-κB status.^39^^,^^40^ Evidence from murine modeling suggests that overexpression of IL1β in mouse forestomach may lead to IL6-dependent intestine-like metaplastic pathologies and tumor-like growths in the junction between the esophagus and the forestomach, which in mice sits midway through the stomach, rather than the higher esophageal cardia junction anatomic location in *Homo sapiens*, canine, and porcine.^41^, ^42^, ^43^ Gastric-specific expression of IL1β in C57BL/6J transgenic mice has long been known to induce gastric dysplasia with marked inflammation and early infiltration of myeloid-derived suppressor cells (MDSCs).^34^ High levels of IL1β are a powerful mediator of acid-suppressive gastritis, with greater potency on acid secretion than proton pump inhibitors (PPIs) such as omeprazole.^37^^,^^41^ Fu et al^21^ recently showed that CD44^+^ cells negative for leucine rich repeat containing G protein-coupled receptor 5 (LGR5) at the murine GEJ were more susceptible to oncogenic transformation to GEJ carcinoma, by inactivation of tumor-suppressor genes *Trp53* and *Rb1*, than their antral cousins. Similarly, KRAS^G12D^- and p53mut-dependent transformation of KRT15^+^ cells at the murine squamocolumnar junction (SCJ) was higher than observed in the regions distal from the SCJ.^22^ This transitional zone may hold precursor cells, at least in mice, that may be predisposed to oncogenic transformations through either inflammatory mechanisms or somatic variation. It is unclear, however, whether these models more appropriately mirror gastric-associated metaplastic transformation linked to gastric cancers rather than the reflux-driven and BE-associated EAC. Nevertheless, these data do suggest a link between localized inflammation and stem cell fate decisions in metaplastic-like transformations of mouse forestomach, but no reports of genomic instability or somatic variations have been reported in these animal models.^41^^,^^42^ Recent evidence pointing toward a gastric cardia origin for the BE lesion, however,^44^ suggests that communication between IL1β released at sites of GERD-wounding in the esophagus and cardia resident stem cell populations, such as Lgr5^-^CD44^+^ cells, warrants critical analysis. In canine reflux models, the presence of squamous barriers to proximal migration of columnar epithelium was not capable of inhibiting columnar metaplasia formation.^45^ Thus, further functional and anatomic investigations are needed with appropriate animal or in vitro models for the improved replication of esophageal adenocarcinogenesis.

## Death or Regeneration: TNFα

**Figure 2:**
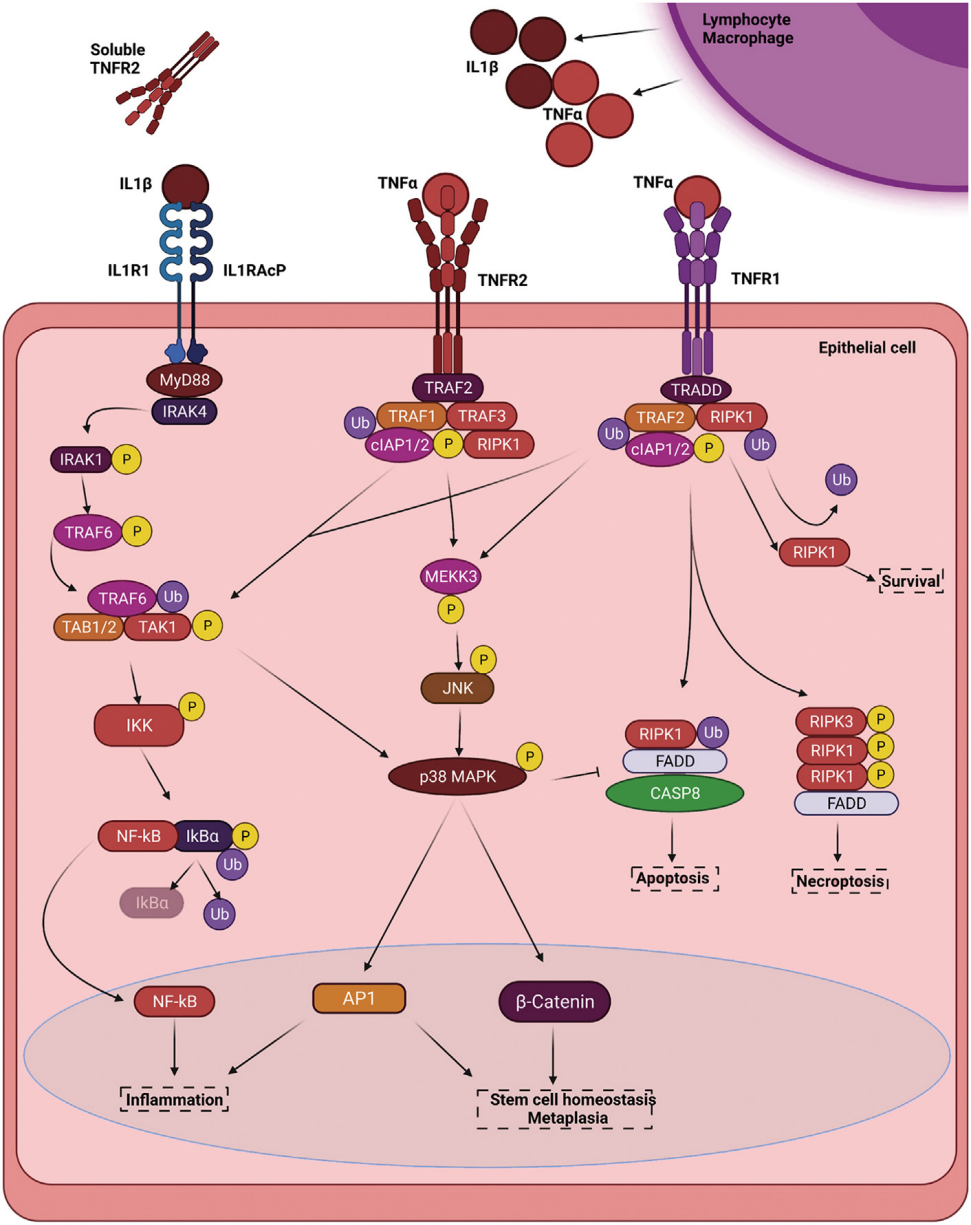
**Overlapping and context-specific signaling in response to IL1β and TNFα.** Dimerization of IL1R1 with IL1RacP transmits the IL1β signal through the MyD88 adapter protein and activation of an IL1-receptor associated kinase (IRAK) cascade. Subsequent phosphorylation of the E3 ligase TNF-receptor–associated factor 6 (TRAF6) induces the polyubiquitin of the TAB1/2 protein and autophosphorylation of TAK1 in complex. This signalosome is then responsible for the activation of the IκB kinase (IKK) complex comprising of IκBα, IκBβ, and NEMO; the phosphorylation and polyubiquitination of IκBα; the release of NF-κB transcription factor; and the subsequent inflammation. TNFα may transmit through TNFR1 and TNFR2 receptors expressed on a wide variety of cells together or independently. The TNFR2 receptor is engaged for inflammatory activation but also may regulate β-catenin activity, as observed in EAC, to impact tissue homeostasis and cell survival signaling through anti-apoptotic gene expression. TNFR1 shows a more restricted expression pattern and regulates death mechanisms through apoptosis, necrosis, or survival in response to TNFα. Both receptors use the TRAF family of E3 ligases to polyubiquitinate downstream targets such as cellular inhibitor of apoptosis protein family members (cIAP1/2), IκBα, and receptor-interacting serine/threonine-protein kinase 1 (RIPK1). Activation of inflammatory signaling through NF-κB is mediated through cIAP1/2-to-TAK1 ubiquitination and the IKK complex activation. In the case of TNFR2, survival and stem cell homeostatic signaling is communicated through a c-Jun N-terminal kinase (JNK)-p38 MAPK activation phosphorylation cascade resulting in AP1 and α-catenin transcriptional activation. TNFR1 activation, on the other hand, dictates survival or death responses through the function of the receptor-interacting protein (RIP) kinase family. The ubiquitination or phosphorylation status of the RIPK1 protein appears to be the predominant dictator of cell fate. Interactions between RIPK1, Fas-associated protein with death domain (FADD), and the CASP8 caspase define an apoptotic response, as opposed to phosphorylation of RIPK1 and its complex with RIPK3 and FADD, of which defines a necroptosis response. Mitigating this signaling is the process of ubiquitination and deubiquitylation of the RIPK1 protein by proteins such as cIAP1/2, resulting in a balance between survival and apoptosis. ILRacP, Interleukin 1 Receptor Accessory Protein; MyD88, Myeloid differentiation primary response 88; TAK, TGF-Beta Activated Kinase; Transforming growth factor-β (TGF-β)-activated kinase 1; TAB1/2, TAK-binding protein 1/2; NEMO, NF-kappa-B essential modulator; CASP8, Caspase 8.

TNFα initially was noted for its cytotoxic effects on tumor cells; however, further studies elucidated both apoptotic and anti-apoptotic functions in the context of inflammatory responses.^55^, ^56^, ^57^, ^58^ The secretion of TNFα is provided primarily by macrophages and follicular dendritic reticulum cells, mast cells, and lymphocytes.^55^^,^^57^, ^58^, ^59^, ^60^, ^61^ The greatest context of TNFα signaling is, to a large extent, provided by its 2 receptors: TNF-receptor (TNFR)1 and TNFR2. Both receptors may facilitate inflammatory activation through transcription factor NF-κB^62^^,^^63^; however, it is broadly proposed that TNFR2 transits a cell survival signal through cellular inhibitor of apoptosis protein 1/2 (cIAP1/2) and BCL2 apoptosis regulator like 2 (BCL2L1 or BCL-XL) activation and TNFR1, inducing a wider array of potential outcomes through the formation of receptor-interacting serine/threonine-protein kinase 1–containing complexes that induce cellular apoptosis and necroptosis. The apoptotic arm of TNF signaling is mediated through TNFR1 and the formation of the death inducing signaling complexes.^56^^,^^64^, ^65^, ^66^, ^67^ The survival component is similarly transmitted through TNFR1, but it involves the ubiquitination-mediated degradation of bound receptor-interacting serine/threonine-protein kinase 1 protein factor^56^^,^^63^^,^^64^ and the activation of a kinase relay that induces survival-mediating pathways such as c-Jun N-terminal kinases, p38, and the critical inflammatory transcription factor NF-κB.^62^^,^^63^ Subsequently, transcription and secretion of potent proinflammatory cytokines—particularly IL1β and IL6—occurs in target cells, be they epithelial, stromal, or of immune origin (Figures 1 and 2).^66^^,^^68^ Although this response traditionally is associated with the initiation of an inflammatory response against microbial pathogens in activated antigen-presenting cells, TNFα-mediated survival also may be hijacked by cancer cell populations.^69^, ^70^, ^71^

The proinflammatory role of TNFα has been implicated in chronic inflammation underlying the BE–high-grade dysplasia (HGD)–EAC progression.^72^^,^^73^ Data from nearly 300 EAC patients showed a high level of circulating TNFα and the associated inflammatory C-reactive protein marker is associated significantly with EAC.^72^ In addition, significantly high levels of soluble TNFα-receptor 2, suggested to originate from uncontrolled shedding of classically membrane-bound TNFα-receptor 2 in EAC (Figure 2), was suggested to result in continuous aberrant inflammatory responses. Intriguingly, TNFα expression was higher in tumor biopsy specimens of EAC patients with pre-existing GERD, suggesting an amplified response mediated by the consistent exposure of esophageal tissue to bile reflux during GERD.^72^ The bile acid deoxycholate, present in the reflux of GERD patients, has been observed to induce both apoptosis and COX2-regulated cell survival in EAC cell lines in a similar fashion to TNFα.^74^ This apoptotic stasis or resistance is likely to further impact TNFα-mediated signaling events during EAC pathogenesis. Indeed, stage-specific induction of IL8 and epithelial-to-mesenchymal transition in EAC cell lines to TNFα treatment have been noted recently.^75^ Comparatively, in other studies, conditioned media from esophageal cancer cell lines was capable of reducing TNFα levels of lipopolysaccharide-activated dendritic cells.^76^ This intriguing juxtaposition requires further attention but also necessitates the significant widening of the commercially available EAC cell lines—which currently consists largely of SKGT4, OE33, and FLO1 for the most part—or the transition into the use of cancer-associated organoid culture systems. The wingless WNT/β-catenin pathway is an essential component of intestinal homeostasis and the intestine-like phenotype and expression pattern underlying BE and EAC development. In familial adenomatous polyposis, mutational loss of function of the *APC* gene results in overactive β-catenin signaling and subsequent promotion of cancer stemness in colonic epithelial cells.^77^, ^78^, ^79^ TNFα levels become progressively higher from metaplasia-dysplasia-carcinoma and, in early data, were observed to up-regulate the c-myc oncogene through β-catenin action independent from NF-κB in esophageal cells (Figure 2).^46^ This evidence suggests that, although TNFα plays a significant role in the maintenance of highly inflammatory microenvironments through NF-κB and its downstream targets, it also directly connects the inflammatory milieu with possible mediators of dysplastic and tumorigenic transformation.^46^^,^^69^^,^^74^ Since these early findings, however, no further connections between TNFα and intestine-like commitment of tissue resident stem cells of the lower esophagus have been noted.

## All Roads Lead to Rome: NF-κB

**Figure 3:**
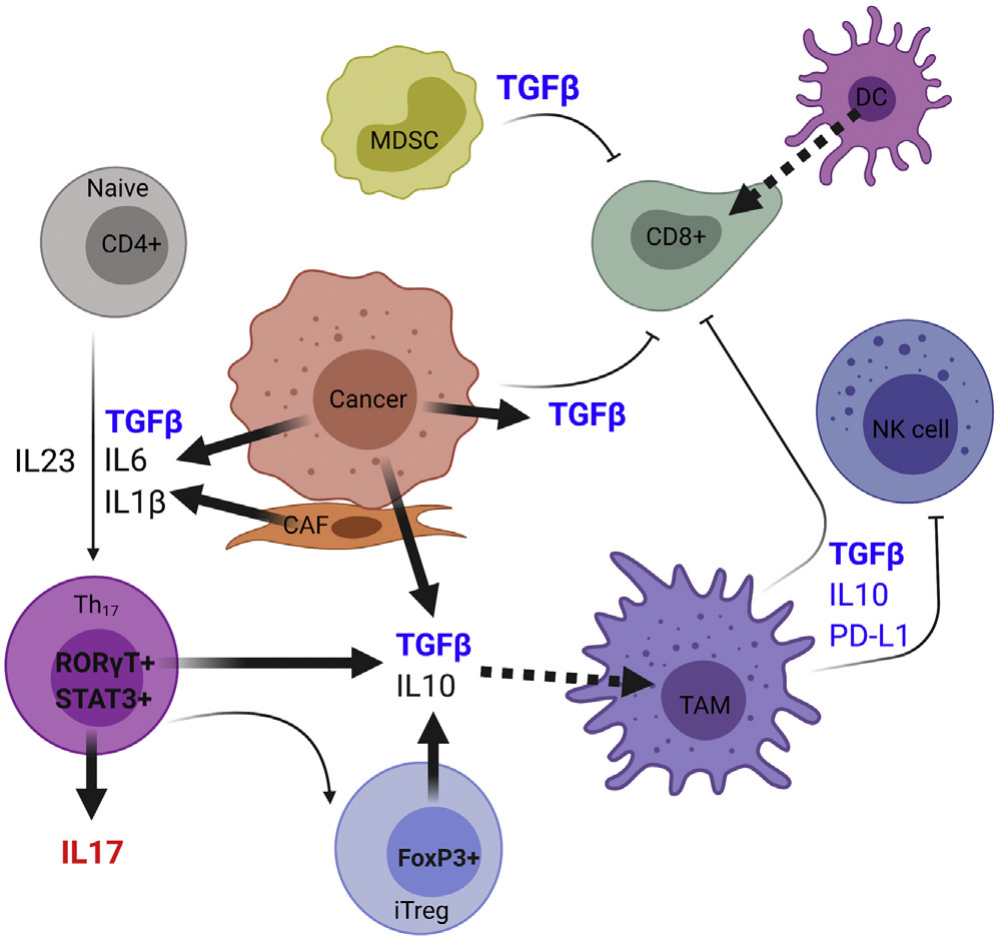
**The multiple sources and immune functions of TGFβ in cancer.** TGFβ, produced by cancer cells and tumor-associated immune cells such as MDSCs, plays a prominent role in supporting the differentiation of naïve immune cells in response to IL6-mediated STAT activation.^98^, ^99^, ^100^, ^101^, ^102^, ^103^, ^104^, ^105^, ^106^, ^107^, ^108^, ^109^, ^110^, ^111^, ^112^, ^113^, ^114^, ^115^ Sources of IL6 include epithelial cancer cells, cancer-associated fibroblasts, and, in the case of treatment-naïve esophageal adenocarcinoma cases, ongoing GERD-mediated injury of the surface epithelium.^49^^,^^50^^,^^116^, ^117^, ^49^, ^50^, ^118^, ^119^, ^120^ Together, IL1β, TGFβ, IL23, and IL6 promote peripheral differentiation of naïve CD4^+^ T cells into inflammatory IL17-producing RORγT^+^ T cells (Th17). Large amounts of immunosuppressive cytokines, particularly IL10 and TGFβ, from M2 TAMs and the tumor itself drive RORγt inhibition, subsequently switching a predominantly inflammatory Th17 CD4+ T-cell population toward a predominantly immunosuppressive inhibitory T-regulatory cell (iTreg) (FoxP3^+^) population.^99^^,^^101^^,^^103^^,^^107^^,^^108^^,^^113^^,^^121^ TAMs, in turn, through the action of TGFβ and PD-L1, can inhibit the activity-infiltrating cytotoxic CD8^+^ T cells and cancer surveillant NK cells. MDSCs also may provide immunosuppressive activities through TGFβ production. DC, dendritic cell; RORγT, retinoid-related orphan receptor gamma t; PD-L1, programmed death-1 ligand 1 (PD-L1).

The NF-κB transcription factor is central to the proinflammatory response, cellular proliferation, and metastasis associated with the development and progression of various human cancers, including EAC.^80^^,^^81^ The canonical pathway involves NF-κB inducing kinase–mediated transmission of receptor signaling to elicit the degradation of the inhibitor of nuclear factor kappa B (IκB) inhibitory protein via the IκB kinase complex, ultimately resulting in the release of the NF-κB p65/p50 heterodimer and higher levels of downstream transcriptional activity.^82^, ^83^, ^84^ Comparatively, the noncanonical pathway consists of the formation of a tumor necrosis factor receptor–associated factor (TRAF)/cIAP1/2/NF-κB-inducing kinase complex, subsequent IκB kinase α phosphorylation, and the activation of RELB proto-oncogene, NF-kB subunit (RelB) heterodimerized with either the p52 or p100 form of nuclear factor kappa B subunit 2 (p52) to form the RelB/p52 or RelB/p100 NF-κB heterodimers.^81^^,^^85^^,^^86^ Although metastatic potential, cell proliferation, apoptotic evasion, and acute inflammatory response have been linked to both pathway arms, the noncanonical pathway may have a more important role in prolonged inflammation owing to its delayed activation through CD40 ligand (CD40L), TNF superfamily member 13b (TNSF13B or BAFF), and TNF superfamily member 12 (TNFS12 or TWEAK) ligands, among others.^84^^,^^85^^,^^87^, ^88^, ^89^ Canonical activation on the other hand can be induced by classic ligand-receptor cytokines such as IL1β, IL8, and TNFα, as defined earlier^90^, ^91^, ^92^, ^93^ (Figure 3). In addition, NF-κB is capable of transcribing pro-IL1β, IL8, and TNFα.^94^, ^95^, ^96^, ^97^

The inflammatory response to reflux stress can be prompted through numerous means, not all of which may be through the caustic damage initiated by gastric bile acids and subsequent innate responses. Nonapoptotic concentrations of bile acids and minor changes in pH have been shown to result in the activation of NF-κB through the degradation of inhibitory proteins such as IκBα in esophageal epithelial cells, subsequently inducing the expression and release of cytokines, such as IL8 and IL6, and the prostaglandin-synthesizing enzyme COX2^38^^,^^39^^,^^47^^,^^80^ (Figure 1). In vitro experiments by Abdel-Latif et al.^38^ in 2004 showed increased activity of NF-κB in esophageal cell lines exposed to a solution of pH 4, mirroring reflux events, in addition to showing significantly lower levels of the NF-κB–suppressive IκBα protein in EAC tumor samples when compared to normal esophageal tissue. This suggests that in EAC tumor cells, the increased levels of active NF-κB may be owed to the increased phosphorylation and subsequent degradation of IκBα in response to prolonged acid damage. This is responsible for the observed increase in IL8 levels throughout the BE–EAC sequence. Thus, at some critical point in the GERD–BE–EAC sequence, constitutive activation of NF-κB and inflammatory proteins, such as IL8, occurs. Subsequent follow-up studies uncovered that the higher level of IL8, IL6, and COX2 expression observed in BE and EAC biopsy specimens was mediated by lower expression of the noncanonical NF-κB regulator tribbles homology 3^39^ (Figure 1). In this study, the mechanism underlying these altered inflammatory responses between cells at either end of the EAC sequence was uncovered using exposure to exogenous deoxycholic acid, a component of bile refluxate. Thus, differing inflammatory IL8 and COX2 signaling observed throughout the squamous BE–EAC sequence may result from cell type–specific losses of tribbles homology 3 expression and function, among other avenues. In summary, hyperactivation of NF-κB through both constitutive proinflammatory signaling and/or loss of negative regulation along the GERD–BE–EAC sequence are common routes to providing a prometaplastic and protumorigenic niche; however, the evidence connecting early activation of NF-κB with the occurrence of genetic instability during dysplasia is less clear.

## Signs of its Passing: Increased Prostaglandins

Prostaglandins are lipid autacoids whose levels increase at sites of wounding or infection and may have both inflammatory and suppressive roles mediated by G-protein–coupled receptor binding in early and late phases of inflammation. Notably, prostaglandins are involved in some of the more visible signs of inflammation such as redness, swelling, and pain. The COX1 and COX2 enzymes together with tissue-specific isomerases convert arachidonic acid into a variety of prostanoids. Although the COX2 synthase, traditionally under the control of NF-κB, mediates the synthesis of many prostaglandin isoforms from arachidonic acid, the most prevalently studied in the context of adenocarcinogenesis is prostaglandin E2 (PGE2).^48^^,^^122^^,^^123^ In early work, both low pH and bile acid exposure was shown to result in COX2 (prostaglandin-endoperoxide synthase 2 *PTGS2*) gene expression in a wide variety of esophageal cell lines.^39^^,^^47^^,^^124^ Interestingly, Jimenez et al^48^ found that the EP2 and EP4 PGE2-receptor subtypes were overexpressed in BE and EAC cell lines, respectively. In addition, they showed that bile acid exposure increased both COX2 and several PGE2-receptor subtypes, suggesting that autocrine signaling of PGE2 may play a role in the BE to EAC sequence.^48^ More recently, PGE2 production by BE tissues were found to be significantly higher than nonerosive reflux disease tissues exposed to reflux.^125^ Although differential functions of PGE2 in BE and EAC mediated by different receptor expression is not well detailed, there are several mechanisms by which PGE2 may promote inflammation and mediate the formation of a protumorigenic microenvironment. Recent work by Moon et al^22^^,^^80^ neatly showed the ability of PPI treatment to suppress Ras/p53-driven tumor formation in the Keratin KRT15^+^ population at the murine SCJ and additionally showed, using both murine and organoid experimentation, that this may be mediated through affects on COX2 activity.

## A Torrential Rain of Cytokines: LIF, IL6, and the STAT3 Axis

Studies have suggested that intrinsic, aberrant STAT3 activation upon BE–HGD–EAC development increases the ability of these cells to evade apoptosis in response to low pH and/or bile acid–rich environments.^40^^,^^116^^,^^126^ IL6, a pleiotropic inflammatory and lineage-specifying cytokine, is produced by both epithelial, stromal, and immune cells alike.^127^, ^128^, ^129^, ^130^ In the classic pathway, IL6 binds to membrane-bound IL6R, initiating a conformational change that complexes with 2 each of IL6, IL6R, and IL6-receptor subunit-β (gp130).^131^ LIF, a cytokine sharing the gp130 receptor subunit with the IL6 family, is expressed in a wide variety of tissue types with functions linked to reproduction, stem cell growth, and embryonic development.^132^, ^133^, ^134^ Both IL6 and LIF signaling lead to nonreceptor tyrosine kinase Janus kinase 3 (JAK3) activation and subsequent dimerization of the STAT3 transcription factor via phosphorylation.^131^ Nuclear translocation of phospho-STAT3 allows promoter binding at target genes such as *VEGF-A, TGFβ,* *Bcl-xL,* and *Mcl-1*.^117^ The consequences of STAT3-mediated transcription include inflammation/immune response, cell survival, metastasis, and angiogenesis, among others, with variations in function between epithelial and immune cells.^40^^,^^49^^,^^116^, ^117^, ^118^^,^^126^^,^^131^^,^^135^

Recent RNA sequencing expression studies from a variety of patient-derived BE, HGD, and EAC tissues has shown a significantly large increase in tissue IL6 expression along the adenocarcinogenesis sequence.^50^ Hyperactivation of IL6/STAT3 signaling in both dysplastic and EAC tissues has been linked to JAK–STAT3–mediated promotion of cancer cell survival in response to bile acid treatment through BCL-XL^116^ and MCL1^126^ levels, which may also be p53-dependent; however, induction of STAT3 signaling also has been observed to be mediated additionally through an epidermal growth factor receptor and Apurinic/apyrimidinic endonuclease-1 (APE1)–mediated axis in a redox-dependent manner.^136^ Although largely a model of murine gastric cancer, the forced overexpression of IL1β in the murine midstomach was observed to promote dysplasia-like lesions through an IL6-dependent mechanism in the stomach.^41^ With recent data pointing toward cardia resident origin of BE stem cells, it may be time to further develop and independently verify this complicated murine cancer model. Divergent from IL6, GERD-associated bile acid–mediated induction of LIF has been observed to be significantly higher in squamous esophageal cells than EAC cell lines,^39^ with a later study confirming significantly higher levels of constitutive LIF messenger RNA expression in HGD/EAC samples compared with healthy esophageal tissues via immunohistochemistry.^40^ In this study, a chronological order for autocrine IL6–STAT3 activation through LIF secretion in EAC cells was determined.^40^ LIF-mediated STAT3 is long known to induce the embryonic stem cell self-renewal transcription factor SRY-box transcription factor 2 (SOX2), an important tissue patterning transcription factor altered during the BE–HGD–EAC sequence.^51^^,^^137^ Recent chromatin immunoprecipitation sequencing analysis of several EAC (n = 20) cells, both of patients (n = 11) and cell lines (n = 9), and ESCC (n = 6) cells showed EAC-specific enrichment of cytokine signaling–related enhancers.^138^ LIF was the most highly enriched EAC-specific cytokine, and its expression is highly dependent on the activity of 4 key master transcription factors that bind to EAC-specific superenhancers: E74 like ETS transcription factor 3 (ELF3), Kruppel like factor 5 (KLF5), ETS homologous factor (EHF), and GATA binding protein 6 (GATA6).^138^ Interestingly, of these 4 EAC-specific master TFs, GATA6 has been implicated as a key driver of the metaplastic transformation of esophageal tissue and survival of oncogenic BE populations,^52^^,^^98^^,^^139^, ^140^, ^141^, ^142^ with somatic variation at the GATA6 locus observed in a significant number of EAC cases according to the cancer genome atlas (TCGA) data. In addition, messenger RNA expression of GATA6 is bile acid–inducible in dysplastic BE and EAC cells, in addition to increased expression along the BE–HGD–EAC progression.^52^ Therefore, the complex interplay between LIF, BE stem cells, and GATA factors in EAC development requires further investigation and may provide a promising target in immunotherapies against EAC.

A higher M2/M1 ratio of tumor-associated macrophages (TAMs) is associated with increased EAC tumor metastatic potential, possibly owing to the immunosuppressive capacities of M2-type TAMs, which drive tumor immune escape.^143^ In vitro studies have shown that both LIF and IL6 are required for the differentiation of peripheral blood mononuclear cells toward an M2 TAM phenotype in studies involving an ovarian carcinoma tumor microenvironment (TME).^144^ Thus, these M2–TAM–promoting cytokines may have potential synergistic actions in the development of EAC and its tumor-immune landscape. Further studies of LIF expression and its relationship to the EAC TME are warranted to fully elucidate LIF’s role in the aberrant IL6/STAT3 epithelial axis, in addition to its influence on the immunologic component of the GERD–BE–EAC sequence. A hypothetical chronological order of events in BE–EAC adenocarcinogenesis mediated by LIF may be as follows: LIF secreted by esophageal squamous and/or inflammatory cells in response to GERD, BE cells become LIF-dependent, GATA6-enriched dysplastic BE cells secrete high levels of LIF, LIF promotes the selection of M2 TAMs, M2 TAMs express high levels of immunosuppressive factors, and dysplastic BE cells progress to prometastatic EAC phenotypes as they evade immunosurveillance. The maintenance of a knife’s-edge balance between inflammatory and immunosuppressive factors in the TME through IL6/STAT3 signaling, and pathologic cell-specific responses to GERD components, may further promote the expression of anti-apoptotic proteins that encourage malignant transformation and clonal selection during EAC development.^116^^,^^126^

## Controlling the Immune Narrative: Transforming Growth Factor-β Family

The cytokine TGFβ is a pleiotropic regulator involved in epithelial and immune homeostasis; immune tolerance; and context-dependent effects on cellular differentiation, development, and tissue homeostasis.^145^^,^^146^ The wider TGFβ protein family consists of 2 main branches with TGFβ 1–3, activin, myostatin, nodal, growth differentiation factor (GDF)-1 and GDF-3, inhibin, and Lefty-1 and Lefty-2 on one arm^146^, ^147^, ^148^; and a second bone morphogenetic protein family branch containing a variety of bone morphogenetic proteins, GDF-5, GDF-6, GDF-7, and anti-Mullerian hormone (AMH/MIS).^146^, ^147^, ^148^ A third and distant branch of the TGFβ superfamily consists of GDF-15, which has been implicated as a driver of metastasis in several cancers, and, more recently, metabolic homeostasis resulting from emerging evidence suggestive of binding to glial-derived neurotrophic factor family receptor α-like.^99^^,^^100^^,^^149^, ^150^, ^151^, ^152^ Dysregulation of TGFβ signaling plays a key role in the development of cancer by promoting both epithelial tumor phenotypes and immunotolerance. Immunologically, TGFβ can promote or suppress the differentiation of various immune cell effectors that are dependent on the modulation of dynamic environmental cofactors such as IL6 cytokine levels.^147^^,^^101^^,^^153^^,^^154^

At steady-state, TGFβ maintains the homeostasis of peripheral CD4+ and CD8+ T-cell levels through a canonical signaling pathway. This is dependent on small mothers against decapentaplegic family member 3 (SMAD3), a receptor-regulated SMAD that mediates the suppression of prostimulatory IL2.^147^^,^^101^^,^^153^^,^^154^ The secretion of proinflammatory cytokines, such as IL6 and IL1β, from activated macrophages, dendritic cells, and neutrophils, bring CD4+ and CD8+ T cells out of a steady-state and toward mature effector phenotypes that now may be directed by localized TGFβ^147^^,^^153^ (Figure 3). For example, TGFβ and high IL6 promotes autocrine production of IL23 from naïve CD4+ T cells in a synergistic and concentration-dependent manner, and this IL23 autocrine signaling has been defined to underlie the formation of proinflammatory type 17 T-helper cell (Th17) lineages^102^^,^^103^^,^^147^^,^^153^ (Figure 3). In a cancer context, TGFβ can suppress the cytotoxic action of cancer surveillant natural killer (NK) cells and CD8+ effector T cells (Figure 3). This mechanism can be explained via the suppressive action of the TGFβ-activated SMAD–ATF1 repressor complex on perforin, granzyme A/B, Fas-ligand, and interferon γ (IFN-γ).^104^ In addition, at the post-translational level, Jun et al^155^ showed a degranulation response prevention in NK cells exposed to TGFβ, preventing the exocytosis of IFN-γ–containing granules required for proper NK-mediated cytotoxic responses. Over the past 30 years, several studies on EAC patient cohorts consistently have shown a significant correlation between patient mortality and the lack of tumor-infiltrating CD8+ T cells and NK cells.^156^, ^157^, ^158^ Recent evidence has shown that this may be owed to the promotion of immunotolerance by an enrichment of inhibitory T-regulatory cells in more advanced tumors, and the possible mechanism by which inhibitory T-regulatory cells promote immunotolerance is through programmed cell death protein 1–mediated apoptosis of CD8+ T cells (Figure 3).^105^ NK cell levels in esophagogastric tumors, including EAC, may be variable and associated with loss of heterozygosity at chromosome 4 and what has become known as a cold immune phenotype defined by low CD8 and IFN-γ–producing cells, supporting the hypothesis of a TGFβ-mediated role in poor outcomes through immunologic means in addition to its epithelial impacts.^159^ In this fashion, interactions between MDSCs and TGFβ is implicated in supporting tumor growth through immunosuppressive functions.^34^^,^^160^ Prolonged inflammatory states, such as those found in BE in the esophagus or inflammatory bowel disease/ulcerative colitis in the colon, allow for the selection of epithelial cells that are more resistant to the growth-suppressive, pro-apoptotic effects of canonical TGFβ signaling.^106^^,^^161^^,^^162^ This protumorigenic selection, combined with the suppression of cytotoxic NK and CD8+ effector T cells induced by TGFβ and chemoattraction of MDSCs, increases the probability of tumorigenesis.^102^^,^^103^^,^^106^, ^107^, ^108^^,^^161^, ^162^, ^163^, ^164^ Developing immunotherapies that promote tumor infiltration by NK and CD8+ T cells, therefore, may be a key future therapeutic strategy.

## The Ties That Bind: Complement Components

Complement component 1q (C1q) is a soluble protein complex acting as a key bridge between innate and adaptive immune responses.^165^, ^166^, ^167^ C1q is secreted primarily by monocyte-lineage cells such as macrophages, and, at steady-state, circulating blood C1q is found at relatively low levels.^165^, ^166^, ^167^, ^168^ During infection and tissue damage, C1q binds to the fragment crystallizable (Fc) region of circulating IgG or IgM antibodies, apoptotic cells, bacterial surfaces, and ligand-bound C-reactive protein.^165^, ^166^, ^167^ This allows for the aggregation of antigen–antibody–C1q complexes to form.^165^^,^^166^ This process, known as opsonization, allows for increased phagocytic efficiency of cells such as macrophages via the classic complement cascade, which ends with the formation of a membrane attack complex that initiates target cell lysis.^165^, ^166^, ^167^ C1q immunoreactivity is increased significantly in the aging brain, and serum levels remain stable up until 40 years of age, after which C1q levels increase progressively with age,^169^ intriguingly tracking the age-related propensity for cancer development.

In the cancer TME, the complement system and its constituent components are proposed to be engaged in the antibody-mediated killing of targeted cancer cells, support chronic inflammatory states, or indeed hamper antitumor responses. Furthermore, some complement components have been observed to support tumor neoangiogenesis. Clinical data from a recent cohort of 134 colon, lung, lymphatic, and blood cancer patients, with circulating C1q levels higher than the 95th percentile, indicated a strong correlation between enriched blood C1q and post-treatment failure.^170^ Increased tumoral C1q recently has been associated with progressive BE-to-EAC adenocarcinogenesis and tumor survival.^40^ In this study, overexpression of C1q subcomponent A chain transcript and protein were found in both BE and EAC patient samples and cell lines. Small interfering RNA–mediated silencing of C1q subcomponent A chain transcription resulted in reduced EAC cell growth through a complement-independent pathway. Supportively, C1q silencing-mediated suppression of in vitro tumor cell growth was restored upon treatment with native human C1q protein. In addition, LIF was proposed as a key regulator in the induction of C1q transcript in EAC cells, suggesting that C1q, in addition to JAK/STAT and spleen tyrosine kinase (SYK)/protein kinase B (PKB or AKT) activation, may be a possible downstream effector of LIF.^40^ Thus, as proposed and shown by Duggan et al^40^, SYK inhibitors, such as fostamatinib, that conventionally target B-cell–driven autoimmune conditions, were capable of inhibiting both STAT and SYK action in EAC cells that reduced their growth. Therefore, fostamatinib may provide a promising method of targeted-treatment against C1q/LIF-enriched EAC tumors.^171^ Interestingly, the Bruton’s tyrosine kinase inhibitor ibrutinib, indicated for B-cell lymphoma and leukemia, is a promising therapeutic against MYC proto-oncogene, bHLH transcription factor (MYC)-amplified EAC tumors, thus further supporting the narrative of innate immune involvement in EAC development.^172^ More recently, Wnt/β-catenin signaling, a critical component of the underlying metaplastic process leading to EAC and a direct regulator of MYC expression, has been shown to be modulated by exposure to C1q in murine aging studies.^173^ This mechanism may explain the hyperactivated Wnt signaling observed in geriatric-associated cancers driven through oncogenic β-catenin activity and high C1q levels.^173^, ^174^, ^175^ Thus, the cellular and protein facilitators of innate immunity may have vital roles in EAC development, given the age profile of EAC patients and the alterations in Wnt/β-catenin signaling in this cancer type.

## The Bugle’s Call: Immune Cell Dynamics in GERD–BE–EAC Transition

Characterizing the population of immune cells infiltrating the esophageal epithelium along BE-EAC progression may provide insight into the aims, resolution, and processing of the local inflammatory or immune response to environmental stressors such as GERD. Studies on the identity of immune cell infiltration in response to GERD and upon BE development are surprisingly scarce; however, they provide some initial insight to the cellular immune microenvironment that potentially shapes and perhaps drives the malignant transition of esophageal epithelium. Current evidence points tentatively towards dynamic populational shifts during this carcinogenic timeline, beginning as a predominantly proinflammatory CD4+ T-cell infiltrate that shifts to one that may be immunosuppressive in late-stage EAC. The proportion of Th1 and Th2 signatures have been used primarily to represent shifts between distinct tissue-immune environments, because Th1 cells primarily secrete IL2 and IFN-γ, whereas Th2 cells secrete IL4 and IL10.^176^^,^^177^ Fitzgerald et al^178^ first investigated Th1/Th2 signatures in esophageal biopsy specimens from normal esophagus, esophagitis, and BE lesions. Cytokines IL1β, IL8, and IFN-γ are indicative (at the time of publication) of a Th1 CD4+ signature; however, in later years, many novel Th subsets have since been investigated but unexplored in the esophageal context. In contrast, BE samples were enriched significantly in IL4 expression compared with esophagitis samples, suggesting a more predominant Th2 signature. In the context of epithelial cell metaplastic transformation, a previous study showed that a predominant Th2 cytokine signature, particularly IL4, has been shown to contribute to the down-regulation of esophageal squamous cell markers and increase in columnar cell signature^179^; however, there now is considerable doubt that BE emerges from a squamous-associated stem cell type. A later study by Kavanagh et al,^180^ using flow cytometry, observed significantly lower overall numbers of CD4+ cells in EAC tissues compared with both esophagitis and BE samples, suggesting that, over time, the presence of activated T cells within tumors is diminished, most likely owing to an immunosuppressive influence. Similar to previous studies, a significantly higher level of IL4-expressing CD4+ cells were observed in BE tissues, but no other markers of a Th2 response reached significance in this study.^180^ Significantly higher production of proinflammatory IL6, IL1β, and granulocyte-macrophage colony-stimulating factor (GM-CSF) than IL10 was observed in ex vivo cultures of BE tissues when compared to normal esophagus with no alterations in IL4 levels. This may suggest that the Th2-type impression of BE is not fully defined yet and requires significantly higher sample numbers and the use of a wider array of multiparametric fluorescence-activated cell sorting (FACS) assays or immune-staining panels. Intriguingly, a similar comparative analysis of the immune cell composition between BE and duodenal tissues found a striking similarity that the investigators suggested was owed to the intestine-like homing signals of BE tissues.^181^ More recent evidence from Lagisetty et al^50^ showed a similar, but more nuanced, trend in immune infiltrate population shifts during the progression from BE, low-grade dysplasia, HGD, and EAC using RNA sequencing and xCell deconvolution analysis (https://xcell.ucsf.edu/). From their analysis, both Th1 and Th2 signatures were significantly greater in EAC vs BE samples; however, unlike Kavanagh et al,^180^ they did not find a significant increase in overall CD4+ infiltration when comparing BE with EAC, only between low-grade dysplasia and HGD.^50^^,^^180^ In addition, there was a significant and step-wise increase in the pro–B-cell signature from BE to EAC. Of the innate immune infiltrate, the presence of eosinophils decreased significantly along BE adenocarcinogenesis while macrophage/monocyte infiltration increased significantly, with a clear M2 signature in EAC compared with HGD.^50^

Although previous studies have shown distinct shifts in immune cell infiltrate composition along BE adenocarcinogenesis, no studies have directly examined if these shifts may be influenced directly by reflux; however, there have been clinical studies examining the relationship between PPIs and eosinophilic esophagitis (EoE) that showed distinct changes in mucosal eosinophil populations post-treatment.^182^^,^^183^ The diagnostic criteria for EoE have been updated to reflect the potential co-existence of EoE and GERD, and that GERD may, in fact, influence the infiltration of eosinophils into esophageal tissue. Indeed, clinical studies have shown PPI treatment reduced the histologic grade of EoE patient lesions over time; however, further studies are required to elucidate the direct influence of changes in GERD status on immune cell infiltrate.^182^, ^183^, ^184^ Recently, Dunbar et al^185^ examined the histologic changes after removal of PPI therapy in GERD patients observing a lymphocyte-predominant inflammatory response, basal cell hyperplasia, with no loss of surface cells at 2 weeks. The investigators thus suggested that pathogenesis of reflux esophagitis may be cytokine-mediated rather than the result of chemical injury.

From these studies, it is unclear how both the innate and adaptive immune infiltrate interact to contribute to the overall lesion milieu. Thus, simultaneous cytometric and cytokine paneling studies of higher-powered cohorts are required to determine this relationship and how they affect the overall immune outcome. Comparing normal squamous esophageal cells with BE and EAC, although still pertinent, may not be as important as comparisons with the GEJ, from which it now is suggested that BE emerges. A recent single-cell RNA sequencing and lineage tracing study proposed that BE arises from the gastric cardia, and that EAC arises from undifferentiated BE cell types.^44^ Thus, investigating the local immune infiltrate within normal gastric antrum or duodenum in addition to the esophagus may be more appropriate comparators when investigating changes in immune cell infiltrate along BE adenocarcinogenesis; however, the inflammatory response of, and damage to, the squamous epithelium in response to GERD still may provide immune signaling supportive of the metaplastic process through promotion of aberrant metaplastic stem cell colonization of the ulcer bed.

## Final Thoughts and Future Directions

Although we have begun to understand and explain some of the potential origins of BE, the mechanisms through which GERD supports its emergence and its transition to adenocarcinoma remains elusive and yet still is guilty by association. It is likely that some aspects of acid and bile acid signaling play an important role in emergent genomic instability, but the impact of chronic or recurrent inflammation at the GEJ cannot be supplanted as a likely driver. The wider immunologic contributors to BE and EAC development have yet to be thoroughly defined, such as those provided by immune cells recruited during GERD, BE, and HGD. These aspects can be studied only from an associative context in clinical samples and have not been as tractable in murine models thus far. Therefore, this review limited its scope to cytokine-mediated signaling in the esophagus and its linkage with the emergence of oncogenic and metaplastic transformation from BE. The majority of the research reviewed herein has been performed using a variety of cell line models of esophageal signaling and tended to lean more upon EAC cell lines than that of normal squamous esophageal cell types. Furthermore, if the true origin of BE is from stem cells within the GEJ then the impact of cytokine signaling upon BE-associated differentiation markers in squamous cells becomes less relevant. Inflammatory signaling in the squamous esophagus, however, is still a key component of BE development whether directly or independently through chemotaxis of immune cells to the GEJ and consequent contributions to metaplasia. Regardless, our research community requires novel solutions, model systems, and platforms for exploring the cellular immunity beneath BE and EAC development that is directed by the cytokine milieu and clearly still an important contributor to metaplasia and dysplasia.

Investment in porcine and canine models in addition to the development of novel platforms may be needed to surmount the obstacles between fundamental basics sciences and translational medicine as it is applied to esophageal pathologies that are poorly modeled in the mouse. Older studies modeling metaplasia in the canine showed a more anatomically correct emergence in response to surgically induced reflux.^7^^,^^186^^,^^187^ These models support the emergence of supposed BE stem cells from the GEJ and obviously are useful for the investigation of inflammatory contributors; however, antibodies and other tools are currently of limited availability for canine and porcine models, and none thus far have been investigated for mutational burden, a critical component of the inherent oncogenic potential of BE. A quicker route to fundamental, human, relevant, and translational discoveries may be garnered from the use of recent organoid technologies capable of mirroring BE and EAC pathologies ex vivo. These approaches, based on cell culturing systems associated with regenerative medicine, allow the propagation of cells and glandular structures from a wide array of pathologies and sufficiently represent the clinical and mutational landscape of the original tissues, at least in early cultures. Under some systems, subculturing over time results in the emergence of dominant clones owing to selective pressure, but this may be dependent on the growth supplements and conditions selected. Such systems have been termed *conditional reprogramming*,^188^ ground state stem cell propagation,^189^ adult tissue–derived organoids,^190^ induced pluripotent stem cell (iPSC)-derived organoids,^191^ spheroids, and air–liquid interface cultures,^192^^,^^193^ and have many useful features and detractions dependent on tissue type and pathology. In the esophageal context, adult-derived organoids of BE and ground state stem cells have shown promise as platforms for the faithful representations of the original tissues but without immune system involvement. Some success has been obtained in maintaining immune cell presence in air–liquid interface cultures of tumor tissues for at least the initial few weeks of culture through the use of IL2-mediated stimulation.^192^^,^^194^ Along these lines, a number of lab-on-a-chip platforms that allow co-culture of immune cells flowing through endothelial-coated channels will be useful in combination with patient-derived organoids. Such systems provide interesting models to examine the influence of cytokines on the maintenance of pathologies such as BE and to examine interactions between immune cells with patient-specific pathologies such as BE or EAC. Importantly, single-cell RNA and DNA sequencing of the heterogenous cell types associated with BE and the resultant cancer may uncover distinct immune profiles or underlying stromal cells with important ramifications that similarly may improve our ex vivo modeling. These approaches critically provide preclinical test beds for potential immune-based therapeutics and traditional biologics before further investment in large animal modeling.

In summary, defining the order of this paradoxic inflammatory pathway underlying BE–EAC may not be as critical as studying how, if, or when it reaches resolution and whether this resolution differs between normal esophagus, columnar BE lesions, dysplastic pathologies, and early cancers. To address these questions, we must rely on, and be informed by, the patient population and their continued engagement with research in addition to the continued development of in vitro organoid technologies and animal models of the BE–EAC transition.
